# Supplementary material for: Learning curve and surgical outcome of robotic assisted colorectal surgery with ERAS program
Source: Sci Rep. 2022 Nov 29;12:20566. doi: 10.1038/s41598-022-24665-w (PMC9709162; doi:10.1038/s41598-022-24665-w)
Supplement: Supplementary file 2 — Supplementary Information 2. [file 41598_2022_24665_MOESM2_ESM.zip › RAL Raw data-Ñ[▒K/051/HS_ñw╝╨░O▒Kñσ.pdf]

正式摘要(電子簽章：000000208288530)<br>W52 / W52 ☐ 英文標題(注意:無英文姓名-請補病人基本檔!)	☐【讀取】憑證	

臺中榮民總醫院
出院病歷摘要

轉入號： 轉出號：

[redacted]

(8)流水編號	
(9)入院日期	110/01/13 (2021)
(10)轉科日期	
(11)出院日期	110/01/18 (2021) 住院天數計 5 日

診斷

- 入院：1.rectal tumor
  2.hypertension
  3.hyperlipidemia
- 出院：1.Submucosal tumor of rectum s/p robotic LAR + loop ileostomy on 2021/1/14
  2.hypertension
  3.hyperlipidemia

主訴

- abdominal pain for about 8 months

病史

- This 55 years old female patient has past history of hypertension, hyperlipidemia. She complained abdominal pain since June 2020. There was no special bowel habbit changed and body weight loss. She came to FungYuan Hospital for help and possitive stool occult blood was found. Sigmoid colon tumor was found in colonoscopy and biopsy showed benign. Repeat colonoscopy was done twice after and no progressive change and definitive malignancy was found in biopsy. She was referred to our hospital for second opinion. Abdominal CT showed sigmoid colon tumor without lymphadenopathy and distal metastasis. Thus, she was admitted for surgical treatment.

體檢發現

- NO OP SCAR...........
  - General appearance :
    - easy looking , conscious clear , without cardiopulmonary distress
    - Vital signs自動帶入date 體溫(BT) 脈搏(P) 呼吸(R) 血壓(BP) 身高(BH) 體重(BW) BMI
      20210113 36.3℃ 71次/分 19次/分 175/100mmHg 158.7cm 75.1kg 29.8
  - HEENT :
    - Eyes: no anemic conjunctiva, no icteric sclera, normal EOM
    - ENT: no otorrhea, no rhinorrhea, no erythematous mucosa, no injected tonsil, no deformity
  - Neck: supple, freely movable, trachea midline,no deformity, no engorged jugular vein , no palpable thyroid or lymph node enlargement
  - Chest:
    - Inspection: Grossly normal, symmetric and fully expansion, no accessory muscle used, no operation scar, no spider angiomata, no gynecomastia
    - Palpation: no palpable mass or nodule, no subcutaneous emphysema, no tenderness
    - Ausculation: clear breathing sound
  - Heart:
    - Percussion: normal heart size
    - Ausculation: regular heart beat, normal S1, S2, no gallop, no murmur
  - Abdomen:
    - Inspection: in
      - flat contour, symetrically, no scar, no stria, no dilated veins, no rashes
    - Ausculation: normoactive bowel sound, no abdominal bruits
    - palpation: soft, no muscle guarding or rigidity, no local tenderness, no rebounding pain, no palpable mass, no palpable pulsation
    - Percussion: no shifting dulllness, no hepatomegaly, no splenomagaly
  -

Peripheral pulsation :	CA	BA	RA	FA	PA	PTA	DPA
Right side :	++	++	++	++	++	++	++/+++
Left side :	++	++	++	++	++	++	++/+++

  - Genito-anus: no tenderness or palpable mass lesion or rectal shelf, normal external genitalia, normal anal tone, brown stool

CRS CONDITION

  - Rectodigital examination: -
  - Colonoscope report: Colonscopy was up to S-colon. Rectal polypoid tumor without significant mucosa abnormal is R/O submucosal tumor, R/O GIST, R/O N
  - CEA: 1.67 ng/ml
  - Imaging studies(Sono , CT of abdomen and chest Films):
    - 檢驗項目 :CT ABDOMEN-SURVEY 報告日期 :1100103
      主 訴 :
      Result: Finding: **For R/I sigmoid colon cancer. 1. Imaging modality and protocol: CT of whole abdomen with IV contrast media and 600cc oral contrast. 2. Tumor location and size: Tumor location: Sigmoid colon Tumor size: Non-measurable. 3. Tumor invasion: Equivocal 4. Regional node metastasis: No 5. Distant metastasis: No 6. Other findings: (1) A 1.2cm renal cyst in left kidney. (2) Liver, pancreas, spleen, gall bladder and right kidney are not unusual. (3) No significant retroperitoneal lymphadenopathy. (4) Bil. visible lower lungs are clear. (5) DJD change of T and L spine. Impression: For suspicious sigmoid colon cancer. No significant regional lymphadenopathy or intra-abdominal metastasis.
  -
    - Lung

手術日期及方法

- 手術日期及方法20210114 Robot Assisted Low Anterior Resection + loop ileostomy

住院治療經過

- After admission, operation with robotic LAR + loop ileostomy was done on 1/14 smoothly. We let her try diet as ERAS protocol. Ostomy rod was removed on POD4. Under the stable condition, she was discharged on 1/18.

合併症

- nil

一般檢查

- 累積報告 :

DATE	TIME	ALB	TP	BIL.T	BIL.D	ALKP	AST	ALT	LDH	CHOL	TG	HDL-C	LDL	UA	NH3	AMY
1100114	1542						17	13								

DATE	TIME	WBC	RBC	HGB	HCT	MCV	PLT	BLST	NEUT	BAND	SEG	LYM	MON	EOS	BAS
1100114	1543	20210		12.4	36.2		274		92.7			5.3	1.9	0.0	0.1

DATE	TIME	NA	K	CL	CA	BUN	CREAT	Mg	P	CRP	ESR	PCT	LIPASE	LACTATE
1100114	1542	138	4.3			8	0.75							
1100118	0923	140	4.5			12	0.78							

特殊檢查

病理報告

其它

出院時情況

- 3.改門診治療

出院指示

藥名	劑量	劑量單位	用法	頻次	數量	數量單位	現況	天數	單位
Ultracet tab	1	TAB	PO	QIDPRN	28	TAB	TH	7	天

出院計劃

主治醫師 2062F 蔣鋒帆 / 住院醫師 2331F 陳凱祥

正式摘要(電子簽章：000000208288530)

W52 / W52

doctorRank:B
